# Treatment Outcome of Tuberculosis Patients Registered at DOTS Centre in Ogbomoso, Southwestern Nigeria: A 4-Year Retrospective Study

**DOI:** 10.1155/2014/201705

**Published:** 2014-09-28

**Authors:** Olarewaju Sunday, Olanrewaju Oladimeji, Folorunso Ebenezer, Babatunde Akintunde, Temitayo-Oboh Abiola, Abdulsalam Saliu, Oluwatoyin Abiodun

**Affiliations:** ^1^Department of Community Medicine, Ladoke Akintola University Teaching Hospital, Ogbomoso, Nigeria; ^2^Liverpool School of Tropical Medicine, Pembroke Place, Liverpool, UK; ^3^Department of Medical Microbiology, Ladoke Akintola University of Technology, Ogbomoso, Oyo State, Nigeria

## Abstract

*Background Information*. Monitoring outcome of tuberculosis treatment and understanding the specific reasons for unsuccessful treatment outcome are important in evaluating the effectiveness of tuberculosis control program. This study investigated tuberculosis treatment outcomes and predictors for unsuccessful treatment outcome in Ogbomoso town, Southwestern Nigeria. *Methodology*. Medical records of all tuberculosis patients registered from January 2008 to December 2011 in 5 Local Government areas, Ogbomoso, Southwestern Nigeria, were reviewed. Treatment outcome and tuberculosis type were categorized according to the national tuberculosis control guideline. Bivariate analysis was used to analyse the association between treatment outcome and potential predictor variables. *Results*. Out of the 965 total TB patients (579 males and 386 females) with mean age 42.4 ± 1.9 years, 866 (89.74%) were categorized as pulmonary tuberculosis and 109 (11.30%) as extrapulmonary tuberculosis. Treatment outcome among total 914 subjects was as follows: 304 (33.26%) patients got cured, 477 (52.19%) completed treatment, 87 (9.52%) died, 9 (0.98%) defaulted, and 1 (0.11%) failed treatment while 36 (3.94%) were transferred out. Higher treatment success rate was associated with those on Category 1 treatment (*P* < 0.05). *Conclusion*. The treatment success rate of tuberculosis patients was high (85.45%) compared to national target. However, certain proportion of patients died (9.52%) and defaulted (0.98%), which is a serious public health concern that needs to be addressed urgently.

## 1. Introduction

Tuberculosis (TB) has reemerged as a major global public health concern since the mid-1980s. Globally, tuberculosis accounted for 1.2–1.5 million deaths (including mortality due to tuberculosis as well as TB and HIV coinfection), with 85% of this occurring in developing countries and 26% in Africa. Possible causes of reemergence are due to rapid increase in poverty, poor living conditions with overcrowding, war, malnutrition, lack of drugs, the chronic problem of underfunding of National Tuberculosis Programmes (NTPs), and nonadherence to programme policies. These factors may contribute to increased transmission of mycobacterium tuberculosis in the community and/or to an increased risk of progression from latent to overt clinical TB [1]. Nigeria is a high burden for tuberculosis (TB). Although the exact burden of tuberculosis in Nigeria is not known, the WHO estimates an incidence rate for all forms of tuberculosis to be 311 per 100,000 populations, incidence of smear positive annually 131 per 100,000 population and prevalence of 546 per 100,000 populations [2]. These figures place Nigeria 4th among the 22 high burden countries in the world [2].

Early diagnosis and adequate treatment of infectious patients with pulmonary TB are necessary to reduce transmission of M. tuberculosis and ultimately to achieve elimination of TB. If TB is detected early and properly treated using a combination of medicines for 6 to 9 months, the patients quickly become noninfectious and are eventually cured. Important challenges for TB control are human immunodeficiency virus (HIV) coinfection and drug resistance [3, 4]. HIV coinfection is the strongest known risk factor for progression of latent TB infection to TB disease [5]. Although HIV coinfection has been shown not to affect the failure rate of TB treatment, high mortality has been reported among HIV-infected TB patients in sub-Saharan Africa [6].

In Nigeria, the National Tuberculosis Control Programme (NTBLCP) was well established body facilitating policy and human development, tertiary care, resource mobilization, and technical support to the state programs in the control of TB, leprosy, and Buruli ulcer while the LGA is the operational level of the program based on the primary health care (PHC) principle (the main thrust of health care in Nigeria). Directly Observed Treatment Short Course strategy based on 5 components, that is, political commitment, case detection by bacteriology, standardized treatment with supervision, effective drug supply, and treatment monitoring, recommended by World Health Organization (WHO) was adopted by NTBLCP to eliminate tuberculosis since 1993 [7]. Moreover, the standardized treatment as recommended by WHO consists of 2-month intensive phase, in which patients take drugs directly under the observation of health care providers, and 4-month continuation phase for new TB cases while the retreatment cases have 3-month intensive phase and 5-month continuation phase. The utility of DOTS has also been demonstrated by Ige et al. who reported 90% sputum conversion rate at second month among 97 patients in a 3-year short course chemotherapy among pulmonary tuberculosis in Ibadan, between April 3, 1995, and April 6, 1998, Ibadan with no relapse after 18 months follow-up while Erhabor et al. at Obafemi Awolowo University Teaching Hospital, Ile-Ife reported a cure rate of 86.1% and compliance rate of 93.8% [8, 9].

Monitoring the outcome of treatment using standardized approach is essential in order to evaluate the effectiveness of the intervention and for comparison. World Health Organization in conjunction with International Union Against Tuberculosis and Lung Disease (IUATLD) provided recommendations on how to evaluate treatment outcomes using standardized categories [10]. This would make it possible to recognize and amend system failures before the incidence and proportion of resistant isolates rise. However, treatment outcome of tuberculosis patients has not been assessed yet in Ogbomoso, Southwestern Nigeria. Therefore, this study aimed to assess treatment outcomes of all TB patients on treatment over 4-year period by reviewing register and case records which will enable us to ascertain the effectiveness or otherwise of this regimen and possible emergence of resistance to antituberculosis drugs in this environment.

## 2. Materials and Method

### 2.1. Study Area

This study was carried out in Ogbomoso, Southwestern Nigeria. It is a city in Oyo State, Southwestern Nigeria, founded in the mid-seventeenth century with a population approximately 645,000 as of 1991. The city is considered one of Nigeria's largest urban centers. The majority of the people are members of the Yoruba ethnic group. Yams, cassava, maize, and tobacco are some of the notable agricultural products of the region.

### 2.2. Study Design

A retrospective analysis of the profile and treatment outcome of all tuberculosis patients registered from January 2008 to December 2012 at DOTS Clinic was conducted. The registration documents reviewed contain basic information such as patient's age, sex, address, tuberculosis type, and treatment outcome. Institutional ethical clearance was obtained from the ethical committee, Lautech Teaching Hospital, Ogbomoso, Oyo State.

### 2.3. Definitions

According to the standard definitions of the National Tuberculosis and Leprosy Control Program guideline (NLCP) adopted from WHO 6, the following clinical case and treatment outcome definitions were used [11].

### 2.4. Pulmonary TB, Smear-Positive

A patient was with at least two sputum specimens which were positive for acid-fast bacilli (AFB) by microscopy.

### 2.5. Pulmonary TB, Smear-Negative

A patient was with symptoms suggestive of TB, with at least two sputum specimens which were negative for AFB by microscopy, and with chest radiographic abnormalities consistent with active pulmonary TB (including interstitial or miliary abnormal images).

### 2.6. Extrapulmonary TB (EPTB)

This included tuberculosis of organs other than the lungs, such as lymph nodes, abdomen, genitourinary tract, skin, joints, bones, and meninges. Diagnosis of EPTB was based on fine needle aspiration cytology or biochemical analyses of cerebrospinal/pleural/ascitic fluid or histopathological examination or strong clinical evidence consistent with active extrapulmonary tuberculosis, followed by a decision of a clinician to treat with a full course of antituberculosis chemotherapy. In all the cases of EPTB, sputum examinations and chest radiographs were used to investigate the involvement of lung parenchyma.

### 2.7. Treatment Outcome

The treatment outcome was divided into seven categories according to NTLCP guideline. These categories were cured (finished treatment with negative bacteriology result at the end of treatment), completed treatment (finished treatment, but without bacteriology result at the end of treatment for those who are smear-positive), failure (remaining smear-positive at five months despite correct intake of medication), defaulted treatment (patients who interrupted their treatment for two consecutive months or more after registration), died (patients who died from any cause during the course of treatment), transferred out (patients whose treatment results are unknown due to transfer to another health facility), and successfully treated (a patient who was cured or completed treatment).

### 2.8. Statistical Analysis

Data collected was analyzed using SPSS statistical package after data cleaning and ensuring data validity through random checks and double entry of data. Frequency data were generated. Both bi- and logistic regression were done in addition to Chi squared testing to demonstrate association between variables of interest. *P* value was set at less than or equal to 0.05 for all inferential statistics having to do with significance tests.

### 2.9. Study Limitation

A large proportion of reported cases (i.e., 486 cases) with missing information were excluded from the study while information on other factors such as education, occupation, housing, and other sociocultural factors that could also affect the treatment outcome was not captured in the register.

## 3. Results

A total of 965 patients with complete documentation were analyzed, age range 1–90 years. Five hundred and seventy-nine (60.0%) were males while 386 (40.0%) were females giving a male to female ratio of 1.5 : 1. The total mean age group for male patients is 43 ± 19 years while that of the female group is 40 ± 17 years and the total mean age group is 42.0 ± 1.9 years (see Table 1).

Likewise, the disease was pulmonary in 854 (88.5%) and extrapulmonary in 111 (11.5%). Nine hundred and seventeen (95.1%) were registered as new cases and put on Category 1 treatment while 48 (4.9%) were registered as retreatment cases and put on Category 11 treatment (see Table 2).

Of the overall 965 patients seen, 477 (33.3%) were cured, 477 (52.2%) had completed treatment given an overall treatment success of 87.5%, 87 (9.52%) died, and 36 (3.94%) were transferred out while 1 (0.01%) failed treatment (see Table 3).

The Local Government area (i.e., Surulere, Ogbomoso South, and Orire LGA) where the patient is taking treatment (*P* = 0.00) as well as treatment category (*P* = 0.00) had significantly associated with favourable treatment outcome (see Table 4).

## 4. Discussion

DOTS is a highly effective and efficient means of managing tuberculosis. The treatment success rate of all tuberculosis cases was 85.5% which is comparable with results from other regions of the world where DOTS strategy is currently being operated. In Cotonou, Benin Republic, 82% and 78% success rates were reported among new and retreatment cases with 1% and 3% failure rates, respectively [12]. In another study, 66.5% cure rate was reported after 7-month short course chemotherapy among smear-positive Rwandan and Burundian refugees [13]. Similarly, 77.2% and 68.3% cure rates, respectively, were reported among smear-positive and smear-negative tuberculosis patients in Sudan [14]. This may be due to full supervision of DOTS Strategy in the treatment centres for 2 months on Category 1 and 3 months for Category 2 during the intensive phase while patients are expected to collect their drugs on monthly basis during the continuation phase.

However, the study also documented unfavourable treatment outcome of 9.52% death, 3.94% transferring out, and 0.01% treatment failure. Other information on responsible factors highlighted from previous studies such as HIV status, occupation, educational status, and poor living conditions was not documented in the record used for this study.

A greater proportion of the TB patients registered within the period were males compared to females. Patients who were mostly affected were within the age range group 20–49 years which is in conformity with findings of Nwachokor et al. and Thomas et al., where predominantly patients below 40 years of age were infected with a peak in the 21 to 30 years of age range. The age groups most affected from this work also conform with global trend in which the middle age groups are most at risk of being infected with tuberculosis. Reasons deduced from this include drug abuse, alcoholism, smoking, and poor living conditions occasioned by unemployment and poverty thus portending grave danger to the society [15]. Also, as observed from the result, there were more than 88.5% and 11.5% cases of pulmonary and extrapulmonary tuberculosis, respectively. This goes to show that pulmonary tuberculosis remains to be the major type of tuberculosis in Ogbomoso by extension Nigeria. This finding is supported by another finding in Ibadan, Southwestern Nigeria, by Cadmus and Salami in Ilorin, North Central Nigeria [16, 17]. Although occurrence of tuberculosis was higher in males than females (1.5 : 1), there remains a great cause for concern on the growing impact of TB as a major cause of morbidity and mortality among women. However, those with favourable outcome had no significant association with gender, age group, type of TB lesion, and new or retreatment TB cases similar to the outcome of the study done by Egbewale et al. in a 4-year review of tuberculosis treatment outcome among 879 TB patients in State Hospital, Osogbo, Southwestern Nigeria [18]. It disagrees with other studies done by Salami and Oluboyo in Ilorin and Gninafon et al. at Cotonou, Benin Republic, where strong association exists with male gender and increasing age [12, 17]. Outcome in our study depends on compliance with therapy and level of monitoring by the community extension workers compared to type of lesion, age, and gender.

There were some limitations to this study: culture for tubercle bacilli was not done; therefore the smear-conversion rate may be underestimated since some bacilli present in the sputum smear may be dead. Also drug susceptibility tests were not done, so we could not assess the degree of resistance to anti-TB drugs. Also, other sociocultural factors and socioeconomic factors such as education, housing, and income that can affect the outcome of patients on treatment could not be retrieved from the register. DOTS is a highly effective and efficient means of managing TB. Efforts should be made to increase treatment centres so as to make them available to patients in developing countries that bear a large burden of the disease. Use of a comprehensive approach, which may include the provision of incentives, transportation, feeding, and others, will go a long way in enhancing DOTS.

## Figures and Tables

**Table 1:** Socio-demographic status of respondents.

Variable (*N* = 965)	Frequency	Percentage
Sex		
Male	579	60.0
Female	386	40.0
LGA		
Surulere	62	6.4
Ogbomoso N	471	48.8
Ogbomoso S	268	27.8
Orire	56	5.8
Ogo Oluwa	108	11.2
Age group		
0–9	39	4.0
10–29	51	5.3
20–29	154	16.0
30–39	185	19.2
40–49	180	18.7
>50	356	36.9

**Table 2:** Distribution of patients in relation to site of disease and treatment category.

Variable	Frequency	Percentage
Pulmonary	854	88.5
Extra-pulmonary	111	11.5
Total	**965**	**100.0**
Cat 1	917	95.1
Cat 11	48	4.9
Total	**965**	**100.0**

**Table 3:** Overall outcome of patients with tuberculosis treatment in Ogbomoso, Oyo State.

Variable	Frequency	Percentage
Cured	304	33.3
Treatment completed	477	52.2
Treatment failure	1	0.01
Dead	87	9.52
Defaulted	9	0.98
Transfer out	36	3.94

**Table 4:** Factors associated with favorable treatment outcome.

Variable	Odds ratio	Confidence interval	*P* value
Treatment Category 1	5.14	2.8–9.3	0.00
Local Government			
Surulere	0.033	0.013–0.80	0.00
Ogbomoso North	0.72	0.42–1.22	0.23
Ogbomoso South	4.77	2.4–9.6	0.00
Orire	5.49	1.23–24.9	0.026
